# Baseline Cardiometabolic, Hepatic and Renal Profile of Infertile Women and Pregnancy Status Following Assisted Reproduction Technology Procedure

**DOI:** 10.1155/tswj/7976886

**Published:** 2026-05-08

**Authors:** Isaac Kofi Adu, Victor Boachie Owusu, Eric K. Amoako, Gordon N. A. Attoh, Emmanuel K. Asiedu, Moses Banyeh, Issah Zabsonre Alhassan, Nafiu Amidu

**Affiliations:** ^1^ Department of Clinical Chemistry, University for Development Studies, Tamale, Ghana, uds.edu.gh; ^2^ Department of Assisted Reproductive Technology, The Chosen Hospital and Fertility Centre, Accra, Ghana; ^3^ Department of Medical Imaging Technology, University for Development Studies, Tamale, Ghana, uds.edu.gh

**Keywords:** cardiometabolic, complete blood count, Ghana, hepatic, infertility, renal

## Abstract

**Background:**

Routinely, the focus of pre–assisted reproduction technology (ART) assessments is ovarian reserve hormones and semen parameters. However, a growing body of research suggests that systemic metabolic health is a critical determinant of reproductive outcomes. The study therefore sought to evaluate disparities in baseline cardiometabolic, hepatic, renal and haematologic factors in infertile women and their pregnancy status following the ART procedure.

**Methods:**

This cross‐sectional baseline assessment with prospective follow‐up study was conducted at The Chosen Hospital and Fertility Centre in Accra, Ghana, from January 2024 to March 2025. The study involved 206 infertile women aged from 28 to 60 years. Venous blood samples were collected and analysed for fasting lipids, complete blood count and renal and liver function, before any ART procedure. The pregnancy status of women was determined after the ART procedure as either positive or negative, and whether their infertility type was primary, secondary or subfertility.

**Results:**

In the multivariable analysis, the chloride level was 0.164 mmol/L higher in women who achieved post‐ART pregnancy with an adjusted odds ratio (95% CI) of 1.179 (1.017–1.366). The proportion of eosinophils was higher in subfertility than in primary infertility (3.7 ± 1.8 vs. 2.3 ± 1.5, *p* < 0.050). In addition, the mean platelet volume (10.4 ± 1.8 vs. 9.8 ± 1.5 vs. 8.3 ± 1.1, *p* < 0.050) and the platelet distribution width (13.3 ± 3.0 vs. 12.4 ± 2.7 vs. 9.9 ± 1.8, *p* < 0.050) were higher in secondary infertility than primary and subfertility.

**Conclusion:**

As a routinely available and inexpensive biomarker, serum chloride could enhance pre‐ART assessment if confirmed in larger prospective studies. The MPV, PDW and eosinophil proportion across infertility types may be indicators of distinct underlying pathophysiological pathways in different infertility diagnoses.

## 1. Introduction

Infertility remains a significant global reproductive challenge, affecting about 48 million couples and 186 million individuals worldwide [1]. Defined as the failure to attain a clinical pregnancy after 12 or more months of regular unprotected sexual intercourse, infertility′s prevalence and incidence are disproportionately high in low‐ and middle‐income countries (LMICs), including sub‐Saharan Africa (SSA) [2]. In SSA, the lifetime prevalence of infertility can be as high as 30% when compared to a global average of 8%–12% [3]. The social implications of infertility in SSA are often severe, encompassing social stigmatisation, marital instability, economic deprivation and profound psychological distress, with women bearing the brunt of these repercussions [4].

In Ghana, infertility is a major reproductive health problem, with studies reporting varying prevalence rates across the country [5, 6]. The aetiologies of infertility are diverse, including tubal factors secondary to untreated pelvic inflammatory disease, anovulatory disorders, endometriosis and male factor infertility [7]. Assisted reproduction technology (ART), including procedures like intracytoplasmic sperm injection (ICSI) and in vitro fertilisation (IVF), represents the most effective treatment for many forms of infertility. However, success rates vary, with a significant proportion of ART cycles failing to result in a clinical pregnancy [8]. This has spurred extensive research to identify predictive biomarkers for ART success, to optimise treatment protocols and counsel patients appropriately.

Routinely, the focus of pre‐ART assessments is reproductive hormones, ovarian reserve markers like antiluteinising hormones, follicle‐stimulating hormones, anti‐Müllerian hormone (AMH) and semen parameters [9]. However, a growing body of research suggests that systemic metabolic health is a critical determinant of reproductive outcomes [10, 11]. The female requires a substantial energy reserve and an optimal metabolic milieu to support the processes of embryo implantation, folliculogenesis and placental development. Consequently, maternal metabolic and nutritional status at the time of conception is increasingly recognised as a key mediator of fertility and early pregnancy sustainability [12].

Dyslipidaemia, characterised by altered levels of fasting lipids, has been linked to adverse reproductive outcomes and poorer ovarian response [10, 11]. The liver is pivotal to steroid hormone metabolism and the synthesis of crucial proteins like albumin and clotting factors, whilst the kidneys regulate electrolyte homeostasis, fluid balance and the excretion of metabolic waste products. Impairments in renal or hepatic function, even within the traditional normal range, could potentially create a suboptimal environment for embryo development and implantation [13, 14]. Despite this possible connection, comprehensive metabolic profiling beyond body mass index and glucose is not routinely integrated into standard infertility protocols in many settings, particularly in resource‐constrained environments such as Ghana.

Therefore, this study is aimed at investigating the association between baseline metabolic health, as determined by the liver, fasting lipids and renal function tests, and clinical pregnancy outcomes following ART procedures amongst Ghanaian women. Additionally, the study seeks to compare these biochemical parameters across infertility groups to elucidate potential metabolic distinctions between these clinical categories.

## 2. Materials and Methods

### 2.1. Study Design and Population

This cross‐sectional baseline assessment with a prospective follow‐up study was conducted at The Chosen Hospital and Fertility Centre, a leading private fertility clinic in Accra, Ghana. The study spanned from January 2024 to March 2025. The study involved a total of 206 infertile women aged from 28 to 60 years. The women were diagnosed with infertility and scheduled for an ART procedure, such as IVF or ICSI, and with a normal menstrual cycle (28–35 days). Women who used medications known to significantly affect lipid or metabolic parameters in the 3 months preceding the study, and those who had pre‐existing diagnosed severe hepatic or renal disease, were excluded from the study. Following the ART procedure, participants were categorised into two groups based on the clinical outcome: the pregnant group (*n* = 134, 65%) and the nonpregnant group (*n* = 72, 35%). Clinical pregnancy was confirmed by both serum hCG and a transvaginal ultrasound scan demonstrating an intrauterine gestational sac at 6–7 weeks of gestation. The women were further categorised based on the type of infertility, into primary infertility, secondary infertility and subfertility.

### 2.2. Sample Size

The minimum sample size was estimated using the Cochrane formula, based on a previously reported prevalence of female infertility of 11.8% in Ghana [15]. A 95% confidence interval and a 5% level of significance were assumed in the calculation. This yielded a minimum required sample size of 139 participants.

### 2.3. Data Collection

#### 2.3.1. Demographic and Clinical Data

A semistructured questionnaire was used to collect demographic and clinical data. Primary infertility was based on the inability to conceive after at least 12 months of unprotected intercourse without any prior conception. Secondary infertility, on the other hand, was defined as the inability to conceive following a previous successful pregnancy. Subfertility was described as a delay in conceiving.

#### 2.3.2. Blood Sample Collection and Biochemical Analysis

Venous blood samples were collected following an overnight fast of 10–12 h and dispensed into gel‐separator and EDTA vacutainer tubes. Blood was collected from each participant on Days 2 or 3 of their menstrual cycle, before the commencement of any ART ovarian stimulation protocol. The blood samples in the gel‐separator tubes were allowed to clot and centrifuged at 3000 rpm for 10 min to obtain serum. The separated serum was aliquoted into Eppendorf tubes and stored at −20°C until batch analysis. Complete blood count and biochemical analyses were performed at the hospital′s accredited central laboratory. The biochemical analysis included fasting lipid levels, liver function tests and renal function tests. All haematological analyses were conducted using the Norma Icon‐5 automated haematology analyser (ELITechGroup, Luxembourg, Belgium). The biochemical analyses were conducted on the BT 1500 automated biochemistry analyser (Biotecnica Instruments S.p.A., Italy). The manufacturers′ recommended reagents, calibrators and quality control (QC) materials and procedures were followed. The QC checks involved three‐level QC material: low, normal and high. The QC results were plotted automatically on the Levey–Jennings chart to ensure there were no violations of the Westgard rules.

### 2.4. Variables

The dependent variables were pregnancy status after ART and infertility subtypes. The independent variables included fasting lipids, complete blood count and liver and renal function variables. Possible confounding variables included sociodemographic (age), clinical (systolic and diastolic blood pressure) and anthropometric (height, weight and BMI) variables.

### 2.5. Statistical Analysis

The data were first collected into an MS Excel spreadsheet for sorting. Data analysis was performed in SPSS Statistical software Version 27.0 (IBM Corp., Armonk, New York, United States). Continuous variables were tested for normality using the Kolmogorov–Smirnov test. Descriptive statistics were presented as *m* *e* *a* *n* ± *s* *t* *a* *n* *d* *a* *r* *d* *d* *e* *v* *i* *a* *t* *i* *o* *n* for parametric data, whilst the categorical data were presented as frequencies and percentages. Group comparisons for continuous variables were performed using the independent samples *t*‐test for two groups or one‐way ANOVA with post hoc test for multiple groups. Univariable and multivariable logistic regression analyses were performed to determine associations between dependent and independent variables. All analyses were two‐sided with a *p* value of < 0.05, considered statistically significant.

### 2.6. Ethical Considerations

The study obtained ethical approval from the Institutional Review Board of The University for Development Studies (Ref. Number: UDS/RB/0019/25). All procedures performed in studies involving human participants were in accordance with the 1964 Declaration of Helsinki or its later amendments. Written informed consent was obtained from all individual participants included in the study. Participation in the study was voluntary and was not tied to one′s political or religious affiliation.

## 3. Results

### 3.1. General Characteristics of the Study Population Stratified by Pregnancy Status

Table 1 summarises and compares the anthropometric and blood pressure variables between ART outcomes. The differences in age, weight, height and BMI between the post‐ART pregnancy status groups did not achieve statistical significance (*p* ≥ 0.050). However, the SBP (*p* = 0.020) and DBP (0.036) were significantly higher in the nonpregnant group than in the pregnant group.

**Table 1:** Comparing the anthropometric and blood pressure variables between ART outcomes.

Variables	Post‐ART pregnancy		*p* value
	Positive	Negative	
Age (years)	41.3 ± 6.9	39.6 ± 6.2	0.228
SBP (mmHg)	121.9 ± 19.2	132.4 ± 23.6	0.020
DBP mmHg)	86.6 ± 11.8	92.1 ± 13.3	0.036
Weight (kg)	72.9 ± 12.4	75.3 ± 13.8	0.369
Height (m)	1.6 ± 0.1	1.6 ± 0.1	0.772
BMI (kg/m^2^)	30.3 ± 7.5	31.2 ± 6.9	0.522

*Note:* The results are summarised as *m* *e* *a* *n* ± *S* *D*, and the differences in data distribution between the groups were determined using the two‐tailed independent Student′s *t*‐test.

Abbreviations: BMI, body mass index; DBP, diastolic blood pressure; SBP, systolic blood pressure.

### 3.2. General Characteristics of the Study Population Stratified by Infertility Type

The pre‐ART age, systolic and diastolic, body weight, height and the body mass index of the women were compared between infertile and subfertile women (Table 2). Although the systolic and diastolic blood pressure, body weight and BMI were higher in the infertility group than the subfertility group, none achieved statistical significance (*p* > 0.050 for all).

**Table 2:** Summary of the anthropometric and clinical data stratified by infertility and subfertility.

Variables	Infertility	Subfertility	*p* value
Age (years)	40.7 ± 6.8	41.1 ± 5.9	0.854
SBP (mmHg)	126 ± 22	121 ± 13	0.490
DBP (mmHg)	89 ± 13	86 ± 9	0.549
Weight (kg)	74.0 ± 13.1	70.5 ± 11.2	0.411
Height (m)	1.6 ± 0.1	1.6 ± 0.1	0.304
BMI (kg/m^2^)	30.9 ± 7.4	27.7 ± 5.3	0.185

*Note:* Results are summarised as *m* *e* *a* *n* ± *s* *t* *a* *n* *d* *a* *r* *d* *d* *e* *v* *i* *a* *t* *i* *o* *n*.

### 3.3. Differences in Baseline Cardiometabolic Factors Between ART Outcome Groups

The cardiometabolic, hepatic and renal function variables were compared between post‐ART outcome groups (Table 3). It was observed that the mean levels of total cholesterol, total protein, albumin, AST, ALT, ALP and chloride were higher in infertile women without pregnancy following ART than in the positive group. On the other hand, the mean levels of triglycerides, GGT, total bilirubin, direct bilirubin, creatinine, urea and sodium were higher in the positive than the negative pregnancy group after ART. However, none achieved statistical significance.

**Table 3:** Comparing hepatic and renal function variables between ART outcome groups.

Variables	Post‐ART pregnancy		*p* value
	Positive	Negative	
Total cholesterol (mmol/L)	5.0 ± 1.2	5.1 ± 1.1	0.602
HDL cholesterol (mmol/L)	1.4 ± 0.4	1.4 ± 0.6	0.657
LDL cholesterol (mmol/L)	2.9 ± 1.1	2.9 ± 1.2	0.914
Triglycerides (mmol/L)	1.3 ± 0.7	1.2 ± 0.6	0.762
Total protein (g/L)	62.6 ± 10.6	65.1 ± 9.8	0.235
Albumin (g/L)	36.9 ± 4.0	37.0 ± 2.9	0.892
AST (IU/L)	19.9 ± 6.4	22.3 ± 7.3	0.096
ALT (IU/L)	16.7 ± 6.5	18.6 ± 8.3	0.197
ALP (IU/L)	67.8 ± 16.2	73.7 ± 23.9	0.144
GGT (IU/L)	30.7 ± 11.8	27.8 ± 10.1	0.221
Total bilirubin (mmol/L)	12.0 ± 3.9	10.9 ± 3.1	0.160
Direct bilirubin (mmol/L)	5.1 ± 2.3	4.3 ± 1.6	0.078
Creatinine (*μ*mol/L)	74.3 ± 18.8	69.1 ± 20.8	0.196
Urea (mmol/L)	4.6 ± 1.3	4.3 ± 1.5	0.510
Potassium (mmol/L)	4.0 ± 0.3	4.0 ± 0.3	0.862
Sodium (mmol/L)	140.0 ± 2.5	139.5 ± 1.9	0.270
Chloride (mmol/L)	99.5 ± 3.0	100.6 ± 4.0	0.107

*Note:* Results are presented as *m* *e* *a* *n* ± *s* *t* *a* *n* *d* *a* *r* *d* *d* *e* *v* *i* *a* *t* *i* *o* *n*.

### 3.4. Association Between Baseline Cardiometabolic Factors and Post‐ART Pregnancy

The association between baseline cardiometabolic and post‐ART pregnancy is shown in Table 4. The results, in the univariable analysis, showed no significant association between baseline cardiometabolic, hepatic and renal factors and pregnancy following the ART procedure. After adjusting for covariates and blood pressure, the chloride level was 0.164 mmol/L higher in the women who achieved pregnancy after ART than those who did not, with an adjusted odds ratio (95% CI) of 1.179 (1.017–1.366).

**Table 4:** Association between baseline cardiometabolic factors and post‐ART pregnancy.

Variables	*B*	OR (95% CI)	*p* value	*B*	AOR (95% CI)^a^	*p* value
TCHOL (mmol/L)	0.094	1.099 (0.774–1.561)	0.598	0.845	2.327 (0.676–8.008)	0.180
HDL (mmol/L)	0.201	1.222 (0.509–2.934)	0.654	−0.413	0.662 (0.173–2.532)	0.546
LDL (mmol/L)	0.020	1.020 (0.708–1.471)	0.913	−0.580	0.560 (0.182–1.723)	0.312
TRIG (mmol/L)	−0.093	0.911 (0.501–1.656)	0.759	−0.561	0.570 (0.214–1.523)	0.262
Total protein (g/L)	0.024	1.025 (0.984–1.067)	0.234	0.029	1.029 (0.981–1.079)	0.238
Albumin (g/L)	0.008	1.008 (0.902–1.127)	0.891	0.033	1.034 (0.892–1.198)	0.660
AST (IU/L)	0.051	1.052 (0.991–1.117)	0.099	0.065	1.067 (0.989–1.151)	0.093
ALT (IU/L)	0.037	1.038 (0.981–1.098)	0.197	0.044	1.045 (0.965–1.132)	0.280
ALP (IU/L)	0.016	1.016 (0.994–1.038)	0.150	0.028	1.028 (0.995–1.062)	0.092
GGT (IU/L)	−0.023	0.977 (0.941–1.014)	0.220	−0.028	0.972 (0.927–1.019)	0.242
TBIL (mmol/L)	−0.083	0.921 (0.820–1.033)	0.161	0.026	1.027 (0.874–1.205)	0.748
DBIL (mmol/L)	−0.194	0.824 (0.662–1.025)	0.083	−0.253	0.776 (0.574–1.049)	0.099
Creatinine (*μ*mol/L)	−0.014	0.986 (0.965–1.007)	0.198	−0.018	0.983 (0.956–1.010)	0.207
Urea (mmol/L)	−0.103	0.902 (0.666–1.222)	0.506	0.086	1.090 (0.751–1.583)	0.650
Potassium (mmol/L)	−0.135	0.874 (0.193–3.947)	0.861	−0.553	0.575 (0.088–3.745)	0.563
Sodium (mmol/L)	−0.101	0.904 (0.756–1.081)	0.269	−0.168	0.846 (0.671–1.066)	0.156
Chloride (mmol/L)	0.100	1.105 (0.977–1.250)	0.112	0.164	1.179 (1.017–1.366)	0.029

Abbreviations: AOR, adjusted odds ratios; *B*, regression coefficients; CI, confidence intervals; OR, odds ratio.

^a^Adjusted for blood pressure and covariates.

### 3.5. Variations in Cardiometabolic, Hepatic and Renal Variables Between Infertility Subtypes

Multiple testing correction test between infertility subtypes regarding cardiometabolic, hepatic and renal variables is summarised in Table 5. No significant differences were found between the infertility subgroups in their cardiometabolic, hepatic or renal variables.

**Table 5:** Comparing hepatic and renal function variables between infertility types.

Variables	Infertility		Subfertility	*p* value
	Primary	Secondary		
Total cholesterol (mmol/L)	5.0 ± 1.2	4.7 ± 0.9	4.9 ± 1.0	0.703
HDL cholesterol (mmol/L)	1.4 ± 0.5	1.4 ± 0.3	1.3 ± 0.2	0.567
LDL cholesterol (mmol/L)	2.9 ± 1.1	2.8 ± 0.8	2.9 ± 1.1	0.974
Triglycerides (mmol/L)	1.3 ± 0.7	1.1 ± 0.5	1.4 ± 0.6	0.485
Total protein (g/L)	63.7 ± 10.6	62.4 ± 10.8	62.0 ± 9.0	0.844
Albumin (g/L)	36.9 ± 3.6	37.6 ± 3.4	36.9 ± 4.6	0.887
AST (IU/L)	20.9 ± 7.2	21.0 ± 5.4	19.4 ± 4.0	0.808
ALT (IU/L)	17.0 ± 7.0	14.7 ± 5.6	22.2 ± 8.0	0.050
ALP (IU/L)	69.1 ± 18.2	77.1 ± 30.5	70.7 ± 19.0	0.530
GGT (IU/L)	29.9 ± 11.6	26.1 ± 8.4	31.3 ± 11.2	0.604
Total bilirubin (mmol/L)	11.7 ± 3.7	11.4 ± 2.8	11.2 ± 4.2	0.925
Direct bilirubin (mmol/L)	4.7 ± 1.9	4.5 ± 2.8	6.0 ± 2.5	0.153
Creatinine (*μ*mol/L)	72.0 ± 20.6	66.2 ± 13.8	81.9 ± 9.9	0.206
Urea (mmol/L)	4.5 ± 1.4	4.2 ± 1.7	4.6 ± 1.3	0.820
Potassium (mmol/L)	4.0 ± 0.3	4.0 ± 0.1	3.9 ± 0.2	0.463
Sodium (mmol/L)	139.8 ± 2.2	139.8 ± 3.2	140.5 ± 2.7	0.660
Chloride (mmol/L)	100.0 ± 3.5	99.3 ± 2.3	99.4 ± 3.8	0.791

*Note:* Results are presented as *m* *e* *a* *n* ± *s* *t* *a* *n* *d* *a* *r* *d* *d* *e* *v* *i* *a* *t* *i* *o* *n*.

### 3.6. Complete Blood Count Variables Between ART Outcome Groups

The complete blood count variables were compared between ART outcome groups (Table 6). The results showed that there were no significant disparities in complete blood count variables between ART outcome groups.

**Table 6:** Comparing complete blood count variables between ART outcome groups.

Variables	Post‐ART pregnancy		*p* value
	Positive	Negative	
RBC (×10^6^/*μ*L)	4.0 ± 0.8	4.0 ± 0.3	0.881
HGB (g/dL)	12.0 ± 2.5	12.8 ± 1.9	0.130
HCT (%)	30.8 ± 7.0	31.0 ± 5.5	0.916
MCV (fL)	74.6 ± 14.2	76.6 ± 11.9	0.462
MCH (pg)	31.2 ± 6.6	31.8 ± 5.0	0.642
MCHC (g/dL)	40.8 ± 8.1	44.3 ± 13.6	0.104
RDW‐CV	16.4 ± 5.4	14.9 ± 1.5	0.123
WBC (×10^9^/L)	5.8 ± 1.4	5.9 ± 1.1	0.801
Lymphocyte (%)	37.8 ± 12.1	34.9 ± 11.6	0.238
Monocyte (%)	8.9 ± 5.3	9.1 ± 2.6	0.895
Neutrophil (%)	48.9 ± 12.5	53.3 ± 12.1	0.085
Eosinophil (%)	2.7 ± 1.7	2.3 ± 1.4	0.245
Basophil (%)	0.07 ± 0.05	0.09 ± 0.08	0.169
Platelet (×10^9^/L)	253.4 ± 81.1	254.6 ± 70.5	0.939
MPV (fL)	9.8 ± 1.5	9.5 ± 1.6	0.362
PDW (%)	12.4 ± 2.8	11.8 ± 2.9	0.304
PCT (%)	0.2 ± 0.1	0.2 ± 0.1	0.617

*Note:* Results are presented as *m* *e* *a* *n* ± *s* *t* *a* *n* *d* *a* *r* *d* *d* *e* *v* *i* *a* *t* *i* *o* *n*.

### 3.7. Association Between Baseline Full Blood Count and Post‐ART Pregnancy

The association between baseline full blood count variables and post‐ART pregnancy is summarised in Table 7. No significant association was observed in the univariable analyses and remained the same after adjusting for covariates and blood pressure measurements.

**Table 7:** Association between baseline full blood count and post‐ART pregnancy.

Variables	*B*	OR (95% CI)	*p* value	*B*	AOR (95% CI)^a^	*p* value
RBC (×10^6^/*μ*L)	0.050	1.051 (0.551–2.004)	0.880	0.825	2.283 (0.113–46.204)	0.591
HGB (g/dL)	0.148	1.159 (0.956–1.406)	0.134	0.413	1.511 (0.702–3.249)	0.291
HCT (%)	0.003	1.003 (0.942–1.069)	0.915	−0.283	0.753 (0.464–1.222)	0.251
MCV (fL)	0.012	1.012 (0.981–1.043)	0.458	0.256	1.291 (0.952–1.752)	0.101
MCH (pg)	0.016	1.016 (0.951–1.086)	0.640	−0.539	0.583 (0.288–1.181)	0.134
MCHC (g/dL)	0.033	1.033 (0.992–1.076)	0.115	0.290	1.336 (0.865–2.064)	0.192
RDW‐CV	−0.174	0.840 (0.687–1.027)	0.090	−0.214	0.807 (0.573–1.138)	0.221
WBC (×10^9^/L)	0.040	1.041 (0.765–1.417)	0.799	0.116	1.123 (0.723–1.744)	0.605
Lymphocyte (%)	−0.021	0.979 (0.945–1.014)	0.238	−0.006	0.994 (0.869–1.138)	0.936
Monocyte (%)	0.006	1.006 (0.922–1.098)	0.894	0.112	1.119 (0.860–1.457)	0.403
Neutrophil (%)	0.031	1.032 (0.995–1.070)	0.089	0.027	1.027 (0.899–1.173)	0.693
Eosinophil (%)	−0.159	0.853 (0.652–1.115)	0.244	−0.126	0.882 (0.611–1.272)	0.500
Platelet (×10^9^/L)	0.000	1.000 (0.995–1.005)	0.938	−0.001	0.999 (0.991–1.007)	0.847
MPV (fL)	−0.126	0.882 (0.674–1.154)	0.359	0.115	1.121 (0.340–3.699)	0.851
PDW (%)	−0.080	0.923 (0.793–1.074)	0.301	−0.094	0.910 (0.475–1.745)	0.777

Abbreviations: AOR, adjusted odds ratios; *B*, regression coefficients; CI, confidence intervals; OR, odds ratio.

^a^Adjusted for blood pressure and covariates.

### 3.8. Differences in Complete Blood Count Between Infertility Subtypes

Multiple testing correction test showed that the eosinophil proportion (%) was higher, whilst the MPV (femtoliter) and PDW (%) were lower in subfertility than primary infertility. It was also observed that MPV (femtoliter) and PDW (%) were lower in subfertility than in secondary infertility (Table 8).

**Table 8:** Comparing complete blood count variables between infertility types.

Variables	Infertility		Subfertility	*p* value
	Primary	Secondary		
RBC (×10^6^/*μ*L)	4.0 ± 0.7	4.1 ± 0.6	4.1 ± 0.4	0.819
HGB (g/dL)	12.2 ± 2.4	12.0 ± 1.6	13.4 ± 1.5	0.285
HCT (%)	30.6 ± 6.5	33.9 ± 6.4	30.5 ± 5.9	0.381
MCV (fL)	74.9 ± 14.0	84.4 ± 9.8	71.1 ± 8.1	0.095
MCH (pg)	31.3 ± 6.4	30.5 ± 4.3	33.0 ± 4.1	0.631
MCHC (g/dL)	42.0 ± 10.7	36.5 ± 7.7	47.1 ± 7.8	0.101
RDW‐CV	16.1 ± 4.8	14.5 ± 1.0	15.5 ± 3.4	0.635
WBC (×10^9^/L)	5.8 ± 1.3	5.9 ± 1.2	6.1 ± 1.8	0.852
Lymphocyte (%)	36.3 ± 12.5	42.4 ± 9.3	36.4 ± 7.3	0.389
Monocyte (%)	9.2 ± 4.9	8.1 ± 1.8	7.7 ± 1.2	0.518
Neutrophil (%)	50.6 ± 13.1	46.1 ± 9.2	52.1 ± 8.4	0.561
Eosinophil (%)	2.3 ± 1.5	3.0 ± 2.1	3.7 ± 1.8^∗^	0.025
Basophil (%)	0.08 ± 0.07	0.07 ± 0.06	0.07 ± 0.06	0.965
Platelet (×10^9^/L)	252.9 ± 76.6	231.6 ± 74.1	279.8 ± 85.9	0.410
MPV (fL)	9.8 ± 1.5	10.4 ± 1.8	8.3 ± 1.1^∗^^#^	0.005
PDW (%)	12.4 ± 2.7	13.3 ± 3.0	9.9 ± 1.8^∗^^#^	0.015
PCT (%)	0.2 ± 0.1	0.2 ± 0.1	0.2 ± 0.01	0.793

*Note:* Results are presented as *m* *e* *a* *n* ± *s* *t* *a* *n* *d* *a* *r* *d* *d* *e* *v* *i* *a* *t* *i* *o* *n*.

^∗^ *p* < 0.05 compared to primary infertility.

^#^ *p* < 0.05 compared to secondary infertility.

## 4. Discussion

The cross‐sectional baseline assessment with a prospective follow‐up study aimed at investigating the association between the baseline cardiometabolic, hepatic, renal, and haematological profile of infertile women and post‐ART pregnancy. The study also investigated the types of infertility amongst infertile Ghanaian women seeking ART services. There were no significant differences in cardiometabolic and hepatic outcomes regardless of post‐ART outcome or the type of infertility. However, the chloride level was higher in the post‐ART pregnancy group than in the negative group after adjusting for covariates. There were subtle but significantly higher mean platelet volume, platelet distribution width, and eosinophil count in primary and secondary infertility than in subfertility.

The lack of significant differences in fasting lipids and hepatic parameters between the pregnant and nonpregnant groups suggests that, within the study population, overt metabolic and hepatic dysfunction or subclinical variations are not primary determinants of ART success. This is consistent with a previous study, which found that baseline fasting lipid levels were not independently associated with IVF outcomes [16]. The results show that for women without a history of severe cardiometabolic or hepatic disease, the functional reserve of these systems is adequate to support the early stages of pregnancy establishment following an ART procedure. This is important for clinical practice, as it indicates that extensive screening of these parameters before an ART procedure, in a generally healthy infertile population, may not yield significant prognostic information regarding the likelihood of pregnancy.

The observed higher baseline serum chloride level, which was independently associated with successful pregnancy following ART, suggests that chloride homeostasis may play an underrecognised role in reproductive outcomes [17]. Chloride may potentially act through its involvement in endometrial receptivity via acid‐base regulation of reproductive tract fluids, chloride channel function or as a systemic indicator of subclinical physiological status [18]. Whilst the precise mechanisms remain speculative, emerging pieces of evidence implicate chloride intracellular channels (CLICs) in endometrial epithelial adhesion during the implantation window, and electrolyte balance is increasingly recognised as critical for gamete transport, embryo development and endometrial preparation [19].

The lack of significant disparities in most of the CBC parameters between the outcome groups following ART suggests that systemic haematological status is not a critical factor in implantation success. This finding is contrary to some previous research that has associated conditions like leukocyte ratios or anaemia with poorer reproductive outcomes [20]. This highlights the complex and multifactorial nature of implantation. It was observed that MPV and PDW were significantly lower in women with subfertility compared to those with primary or secondary infertility. MPV and PDW are recognised markers of platelet activation and reactivity, where higher values are often associated with a prothrombotic state [21]. A previous study showed that an activated platelet state was linked to impaired endometrial receptivity and placentation, potentially contributing to infertility and pregnancy loss [22]. It is plausible that subfertility, often involving milder or ovulatory factors, may be linked to a different haematological profile compared to the more defined aetiologies of primary or secondary infertility. This could reflect differing underlying inflammatory or immune mechanisms. The finding of lower MPV and PDW in subfertility is counterintuitive and requires further investigation.

Furthermore, the study found a significantly higher eosinophil count in subfertility compared to primary infertility. Eosinophils are granulocytes traditionally associated with allergic responses and parasitic infections, but they are now recognised as playing a role in tissue remodelling and immune regulation in various physiological and pathological contexts, including the endometrium [23]. However, their precise role in human fertility is ill‐defined. The elevated count in subfertile women could suggest a unique, low‐grade inflammatory or immune‐mediated process distinct from other infertility types. Given Ghana′s location in a tropical region where parasitic infections are more prevalent, these findings warrant consideration of region‐specific environmental or infectious factors that may subtly influence the immune milieu of the reproductive tract [24].

The study′s strengths include its settings. The study provides vital but rare data regarding baseline nonhormonal biochemical variables of infertile women and how these relate to the outcome following ART procedure in Ghana. In addition, blood was collected for laboratory analysis before the initiation of any form of treatment. This avoided or reduced possible errors and ensured that the results reflect the true status of the women at baseline. However, the cross‐sectional baseline assessment with a prospective follow‐up design, which allows for the identification of associations, did not allow for causal relationships of inference. Moreover, the study was conducted in a single private centre in Ghana, which may not be representative of the broader population.

## 5. Conclusion

As a routinely available and inexpensive biomarker, serum chloride could enhance pre‐ART assessment if confirmed in larger prospective studies. The finding of disparities in MPV, PDW and eosinophil counts across infertility types opens a new opportunity for inquiry. These markers may not be associated with ART success, but rather indicators of distinct underlying pathophysiological pathways in different infertility diagnoses. Future prospective, longitudinal studies with larger, multicentre cohorts are warranted to validate these findings.

## Author Contributions

Isaac Kofi Adu, Moses Banyeh and Nafiu Amidu: conceptualisation and methodology; Nafiu Amidu and Moses Banyeh: supervision, administration and validation; Isaac Kofi Adu, Victor Boachie Owusu, Eric K. Amoako, Gordon N. A. Attoh, Emmanuel K. Asiedu and Issah Zabsonre Alhassan: experimentation, data collection, data curation, statistical analysis and writing draft.

## Funding

No funding was received for this manuscript.

## Disclosure

All the authors reviewed the draft manuscript and approved its content.

## Consent

All participants offered written informed consent before enrolment in the study. The study was voluntary, and a participant could withdraw at any stage. Participants gave their consent for the publication of the study findings.

## Conflicts of Interest

The authors declare no conflicts of interest.

## Data Availability

The data supporting the findings of this study are available through a responsible request from the corresponding author.
